# Discovering the chloride pathway in the CFTR channel

**DOI:** 10.1007/s00018-019-03211-4

**Published:** 2019-07-20

**Authors:** Bianka Farkas, Hedvig Tordai, Rita Padányi, Attila Tordai, János Gera, Gábor Paragi, Tamás Hegedűs

**Affiliations:** 1 Department of Biophysics and Radiation Biology, Semmelweis University, Budapest, Hungary; 2 Faculty of Information Technology, Pázmány Péter Catholic University, Budapest, Hungary; 3 MTA-SE Molecular Biophysics Research Group, Hungarian Academy of Sciences, Budapest, Hungary; 4 Department of Pathophysiology, Semmelweis University, Budapest, Hungary; 5 Department of Medical Chemistry, University of Szeged, Szeged, Hungary; 6 MTA-SZTE Biomimetic System Research Group, Hungarian Academy of Sciences, Szeged, Hungary; 7 Institute of Physics, University of Pécs, Pecs, Hungary

**Keywords:** Cystic fibrosis, ABCC7, Chloride channel, Structure, Molecular dynamics

## Abstract

Cystic fibrosis (CF), a lethal monogenic disease, is caused by pathogenic variants of the CFTR chloride channel. The majority of CF mutations affect protein folding and stability leading overall to diminished apical anion conductance of epithelial cells. The recently published cryo-EM structures of full-length human and zebrafish CFTR provide a good model to gain insight into structure–function relationships of CFTR variants. Although, some of the structures were determined in the phosphorylated and ATP-bound active state, none of the static structures showed an open pathway for chloride permeation. Therefore, we performed molecular dynamics simulations to generate a conformational ensemble of the protein and used channel detecting algorithms to identify conformations with an opened channel. Our simulations indicate a main intracellular entry at TM4/6, a secondary pore at TM10/12, and a bottleneck region involving numerous amino acids from TM1, TM6, and TM12 in accordance with experiments. Since chloride ions entered the pathway in our equilibrium simulations, but did not traverse the bottleneck region, we performed metadynamics simulations, which revealed two possible exits. One of the chloride ions exits includes hydrophobic lipid tails that may explain the lipid-dependency of CFTR function. In summary, our in silico study provides a detailed description of a potential chloride channel pathway based on a recent cryo-EM structure and may help to understand the gating of the CFTR chloride channel, thus contributing to novel strategies to rescue dysfunctional mutants.

**Electronic supplementary material:**

The online version of this article (10.1007/s00018-019-03211-4) contains supplementary material, which is available to authorized users.

## Introduction

Cystic fibrosis is a monogenic disease associated with high mortality and is caused by mutations affecting the CFTR (cystic fibrosis transmembrane conductance regulator also known as ABCC7) protein, a chloride channel in the plasma membrane of epithelial cells. In the presence of mutations a lack of functional CFTR expression in the apical membrane is observed resulting in imbalanced salt and water homeostasis [1]. The most frequent mutation in patients with cystic fibrosis (CF) is the deletion of F508 (F508del) impairing both protein folding and function [2]. Recent clinical trials are promising, and combinations of several drugs have been reported to act on the affected steps of CFTR biogenesis, trafficking and function [3]. For understanding the effects of mutations and drugs on CFTR, the knowledge of high-resolution structure and dynamics of the channel in the context of gating is essential. This information is also crucial for the development of more potent treatment modalities correcting the effect of F508del and other mutations.

CFTR (ABCC7) is the member of the ATP binding cassette (ABC) superfamily. It consists of two transmembrane domains (TMD1 and TMD2), each consisting of 6 TM helices, two nucleotide binding domains (NBD1 and NBD2), and a disordered segment, called regulatory (R) domain [4, 5]. The R domain phosphorylation is a prerequisite for channel gating. The N-terminus, also called Lasso/L0 region, plays a role in the functional expression of CFTR through its intramolecular and intermolecular protein interactions [6, 7]. The ATP binding sites are formed by Walker A and B motifs in one of the NBDs and the ABC signature motif in the other NBD. The site with the Walker motifs from NBD1 is non-hydrolytic. An additional intriguing property of NBD1 is the presence of a roughly 40 amino acid long regulatory insertion between the β1 and β2 strands [8]. This insertion destabilizes NBD1 and is highly flexible, thus it is not visible in structures [9]. The conformation changes in the NBDs are transmitted to the TMDs via the so-called intracellular loops that are not loops in a structural sense, since they are formed by the intracellular continuation of two neighboring TM helices (TM2/3, TM4/5, TM8/9, and TM10/11) connected by a small helix (Fig. S1). These four short helices are called coupling helixes (CH1-4), as they interact with the NBDs and couple the ATP dependent conformational changes to the transmembrane domains [10]. Importantly, two TM helices from the two TM domains cross over to the opposite NBD in a domain swapping-like conformation [10]. For many years, only the structure of NBD1 had been known at atomic resolution [11]. After publishing the first full length ABC protein structure, several CFTR homology models have been built and some of their properties have also been experimentally confirmed [10, 12–15]. Major aspects of the homology models have been reinforced by recent cryo-EM structures, which also exhibit features unpredictable by homology modeling, for example the kink in TM8 [16–19]. In addition, the cryo-EM structures by Chen et al. and Fay et al. provide interesting, albeit low resolution information on R domain localization and on extracellular loop 4 (EL4) conformation [16, 20]. EL4 is the longest among the extracellular loops, is glycosylated, and is not visible at high resolution in any of the structural models. The CFTR structure has been determined in the absence of ATP and also in its active state, when ATP molecules were bound to the phosphorylated protein.

Although cryo-EM structures were determined in various conformations, understanding of channel function is still lacking. To overcome the limitations of static structures, several molecular dynamics studies have been performed. Tordai et al. performed simulations with the apo zebrafish CFTR structure, which was determined in the absence of ATP and exhibited separated NBDs [21]. In this study, it was suggested that this conformation is not highly populated under physiological condition, since closure of the NBDs could be observed even in short simulations. Corradi et al. have also detected this closure and membrane defects in the bilayer around TM8 [22]. They found that the position of the kinked TM8 is stable in long simulations using either the apo or the ATP-bound conformation. In a recent paper, Hoffmann et al. combined their ATP-bound CFTR homology model with metadynamics simulation to describe conformational changes associated to gating, since even the cryo-EM structure determined in the active form did not exhibit an open pathway for chloride ions [6]. Chin et al. also performed long molecular dynamics (MD) simulations with an ATP-bound human CFTR homology model, based on the ATP-bound zebrafish structure and focused on the lipid interactions of the CFTR protein [23]. This and other studies showed that lipid interactions are essential for high CFTR ATPase activity, which hydrolytic activity had been demonstrated and thought to be very low compared to other ABC transporters [1, 16, 23, 24].

Since none of the CFTR structures determined in active state demonstrate an open chloride channel and none of the in silico studies characterized in detail the chloride passage through the protein, in the present study we performed MD simulations to generate open conformations with the intention of identifying chloride pathways, and characterizing the interaction of CFTR protein with chloride ions.

## Methods

### Structure preparation

We used the cryo-EM structure (PDBID: 5W81) of phosphorylated, ATP-bound zebrafish CFTR (zCFTR) [17]. Residue indices in the figures are renumbered according to the human CFTR indexing. TM helix numbering is presented in Fig. S1.

### Molecular dynamics (MD)

The starting structure was oriented along the membrane normal with the help of the OPM database and server [25]. MD simulations were performed using the GROMACS 2018 program package in which the CHARMM36m force field and the TIP3P water model were selected [26, 27]. The CHARMM-GUI web interface was employed to build a lipid bilayer environment around the protein model, as well as to generate input files for energy minimization, equilibration steps (NVT, NPT), and production run [28–30]. Input MDP files can be downloaded from http://cftr.hegelab.org. During system preparation, the following options were applied: a homogenous lipid bilayer was built from POPC (1-palmitoyl-2-oleoyl-sn-glycero-3-phosphocholine) molecules and 150 mM KCl was set for physiological liquid environment; grid information for PME (Particle-Mesh Ewald) electrostatics was generated automatically, and NPT ensemble was selected with constant number of particles (*N*), pressure (*P*) of 1 bar, and temperature of 310 K. Parameters for ATP were provided by Komuro et al. [31]. All the structures were energy minimized in the first step using the steepest descent integrator (converged when force < 1000 kJ/mol/nm). The fast smooth PME algorithm was used to calculate electrostatic interactions and LINCS to constrain bonds. The equilibration procedure was performed 22 times to generate inputs for independent simulations with different starting velocities and this was followed by 35 ns long production simulations. A simulation with open conformations and five other randomly selected ones were extended to 100 ns long simulations to increase the possibility of observing open conformations. RMSD (root mean square deviation from the initial structure) and *R*_g_ (radius of gyration) plots indicated that the simulations with the near-atomic resolution zCFTR structural model (3.37 Å) were stable (Fig. S2). A brief summary of simulations can be found in Table S1. During the analysis (e.g. for RMSD and contact map calculations), GROMACS tools, the MDAnalysis package [32] and in-house Python scripts were applied.

### Metadynamics

A snapshot from the equilibrium trajectory with open conformations was selected as a starting structure for further studies. According to the MD simulation, two chloride ions (chloride #1 and #2) entered the interior region, and our starting structure selection was based on the position of chloride #2, which approached the bottleneck region closer than chloride #1. This starting geometry was equilibrated for 25 ns with a position restraint on chloride #2 and the final state was used as input for a well-tempered metadynamics simulation. A distance based collective variable (CV) was biased in the metadynamical running, namely the distance between chloride #2 and the center of mass (COM) of four Cα atoms (residues 96, 348, 932, and 1149; human indexing: 95, 347, 924 and 1141). Focusing on the interior region, an extra constraint was introduced for the CV variable to restrict its value between *z* = 5 Å and *z* = 20 Å. This approach prevented the escape of the chloride ion towards the intracellular or the more distant extracellular regions. Finally, an angle restraint provided further control of the ion movement to keep it in the region of interest. Namely, we considered three points including the COM of the POPC P atoms in the extracellular leaflet, the COM of the POPC P atoms in the intracellular leaflet, and the actual position of the chloride ion. Connecting these three points in this order, an angle was defined and it was kept smaller than 80° during the simulation (Fig. S3). The well-tempered metadynamics simulation was running for 600 ns using the GROMACS augmented with the PLUMED 2.3 package [33]. The free energy surface (FES) for chloride #2 was projected onto the *x*/*y* and *x*/*z* planes using PLUMED tools. Convergence of the metadynamics simulation is demonstrated in Fig. S4 and input files can be accessed at http://cftr.hegelab.org.

### Channel detection

As the first step, the Hole2 program [34] was used to identify conformations with pathways sufficient for chloride passage in the transmembrane region. All frames from every simulation were screened. A conformation was considered as open in the TM region if the radius of the detected pore was larger than 1.8 Å (radius of the chloride ion) in the membrane region, defined by the COM of two groups of Cα atoms located approximately at the level of the intracellular lipid head groups (a.a. 190, 250, 992, and 1047; human indexing: 189, 223, 1018, 1023) and at the level of the extracellular head groups (a.a. 218, 223, 1018, and 1023 human indexing: 217, 222, 1010, and 1015). In the second step, the conformations open in the TM region were subjected to the Caver program [35], which allows a more sophisticated tunnel search at the expense of speed. The starting point of this search was defined by the COM of two Cα atoms (190 and 990; human indexing: 189 and 982). The residues of the transmembrane domains (a.a. 1–384 and 844–1181; human indexing: 1–383, 846–1173) were exclusively used in the calculation. The cutoff radius was set to 1.8 Å. The parameters of shell radius and shell depth were set to 6 Å and 3 Å, respectively. The clustering threshold was set to 4.5 Å. The “one tunnel” setting in the snapshot option was turned off. The tunnel residues were calculated using the default contact distance of Caver (3.0 Å). All other parameters were set to default values. Several tunnels were detected from the starting point ending at either the intracellular or extracellular sites. Conformations containing at least two tunnels connecting the intracellular and extracellular spaces were selected for further analysis. The pair of tunnels with the lowest scores (tunnel cost parameter) were set as the most probable open pathway. Hole2 and Caver input parameters can be downloaded from http://cftr.hegelab.org and their output was analyzed using in-house Python scripts. The interaction of amino acids with Caver spheres and chloride ions were calculated using MDAnalysis. Clustering the channels in the open conformations was performed based on atom positions of the channel-lining residues (criterion: Ward’s method). The frames were aligned to the initial structure for analysis and the *z* = 0 corresponds to the bottom of the simulation box.

### Network analysis

The NetworkView plugin of VMD was applied to build, analyze, and display the network based on the pairwise correlated motions of amino acids [36–38]. MDTraj [39] was used to convert a part of the trajectory (from 80 to 100 ns) in a suitable format for Carma [40], which calculated the correlation in Cα motions. The *subopt* code from the authors of NetworkView plugin was run to calculate optimal and suboptimal paths between specific residues. The configuration files and parameters can be found at http://cftr.hegelab.org.

### Visualization

Structures are visualized using PyMOL (The PyMOL Molecular Graphics System, Version 1.8.4 Schrödinger, LLC) or VMD [36]. Figures were generated by Matplotlib [41].

## Results

### Identification of open channels suited for chloride conductance

The determination of CFTR structure in its active state with the phosphorylated R domain and the ATP-bound nucleotide binding domains (NBD) enabled us to examine the structural background of chloride conductance. However, the cryo-EM structure of the phosphorylated and ATP-bound zebrafish CFTR structure (PDBID: 5W81) discloses no pathway suitable for chloride passage [17]. To find CFTR conformations with open channels, we performed equilibrium molecular dynamics (MD) simulations (*n* = 22) with different initial velocities using this zCFTR structure (Table S1). We used the “open conformation” expression for structures with a radius larger than 1.8 Å along the entire pathway, which geometry can ensure chloride conduction. Firstly, we identified conformations with an open transmembrane region using the Hole2 program [34]. Subsequently, the resulting structures were filtered for continuous channels between the intra- and extracellular spaces by the Caver code [35]. Among the 22 equilibrium simulations, only a single trajectory (see detailed below) exhibited conformations with open channels.

**Fig. 1:**
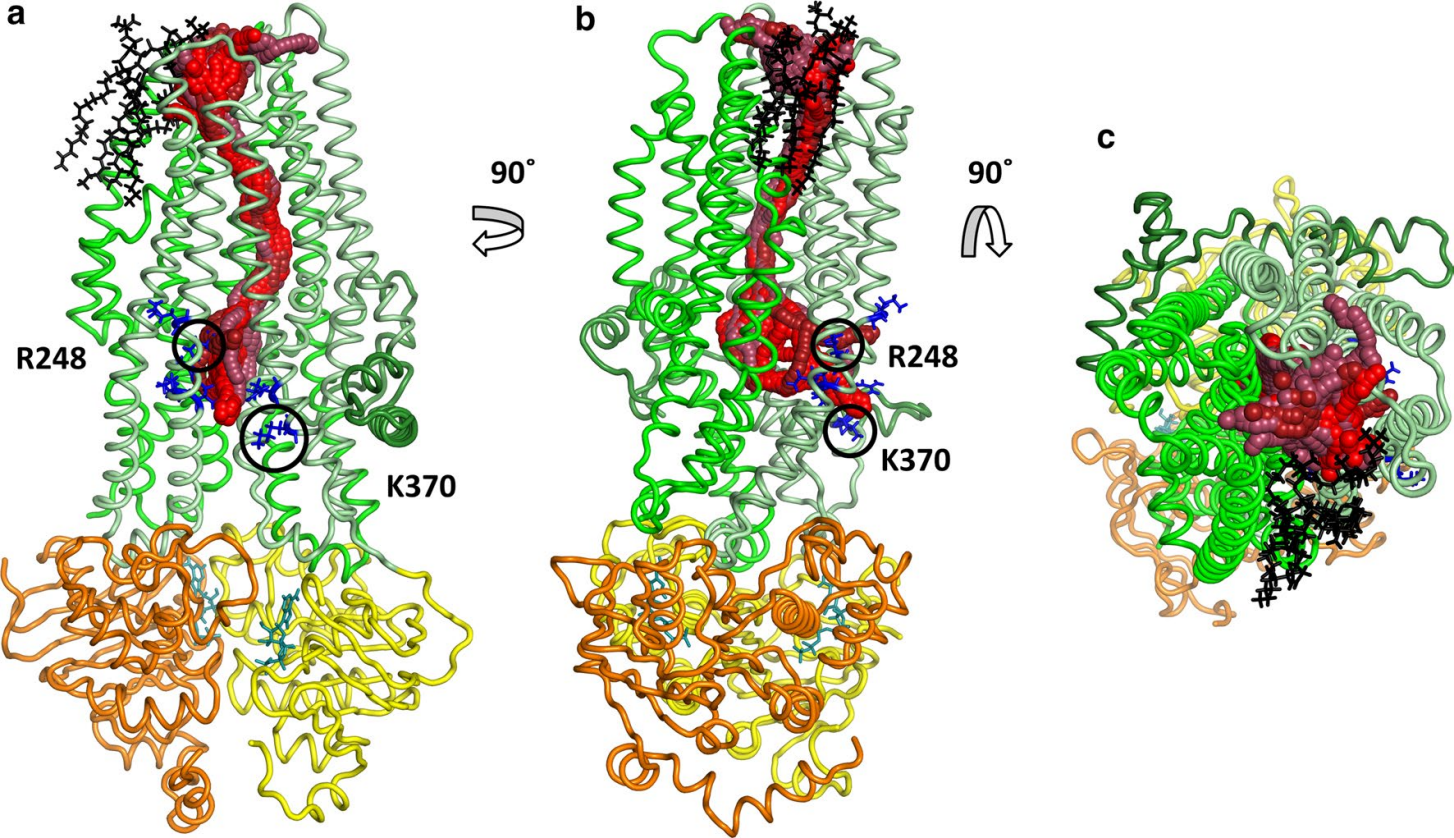
Channels with extracellular and intracellular ends. Tunnels suitable for chloride conductance were identified in one case out of 22 equilibrium simulations with the ATP-bound CFTR structure (PDBID: 5W81) using Caver. The channels with top scores (spheres with red colors) were clustered based on the atom positions of the channel-lining residues. Intracellular residues with positive charges at the TM4/6 entry site are labeled with blue sticks. Lasso/L0 region, TMD1, TMD2, NBD1, and NBD2 domains are marked with dark green, pale green, green, yellow, and orange, respectively. Lipid molecules lining the pore in the region of TM8 are shown by black sticks. **a**, **b** Side views from the bilayer hydrophobic core. **c** A top view from the extracellular space

Concerning the full trajectory, a small portion of the total frames (54/10,000) contained channels having simultaneously open geometry at both sides. Importantly, opening events were not grouped around a given time point, but distributed evenly over the 100 ns simulation time (see below). In order to compare the open conformations, we performed clustering on the channel-lining residues. An amino acid was selected as a pore facing residue if the distance between any of its atoms and the Caver channel spheres was smaller than 3 Å (Cavers’s default value). The tunnel clustering was performed using the pairwise RMSD of the channel-lining residues as a distance measure. Tunnels in all the resulted clusters were highly similar in the TM region and diverged only in the intracellular parts (Fig. S5). While differences in the intracellular ends could be observed, these tunnels were all open between TM3, TM4 and TM6 around amino acids at position K190, R248 (K in zCFTR), G366 (R in zCFTR), K370 (human CFTR residue indexing is used throughout the paper) (Fig. 1). A second, symmetrical opening with lower Caver scores was also apparent in some of the simulations, between TM9, TM10 and TM12 around amino acids G971 (R in zCFTR), R975, Q1035 (R in zCFTR), K1041, R1048, R1158, R1162 and K1165 (Fig. S5). The physiological relevance of these two intracellular gates, which are surrounded by positively charged residues, is supported by experiments [42].

Interestingly, some of the conformations exhibited a large opening to the hydrophobic part of the membrane bilayer in the TM8 region (Fig. 1) indicating that lipid molecules can interact with the pore residues. In MD simulations, we observed POPC molecules that could even enter the channel. As a consequence, the channel became closed by the hydrophobic lipid tail that can prevent the chloride passage.

**Fig. 2:**
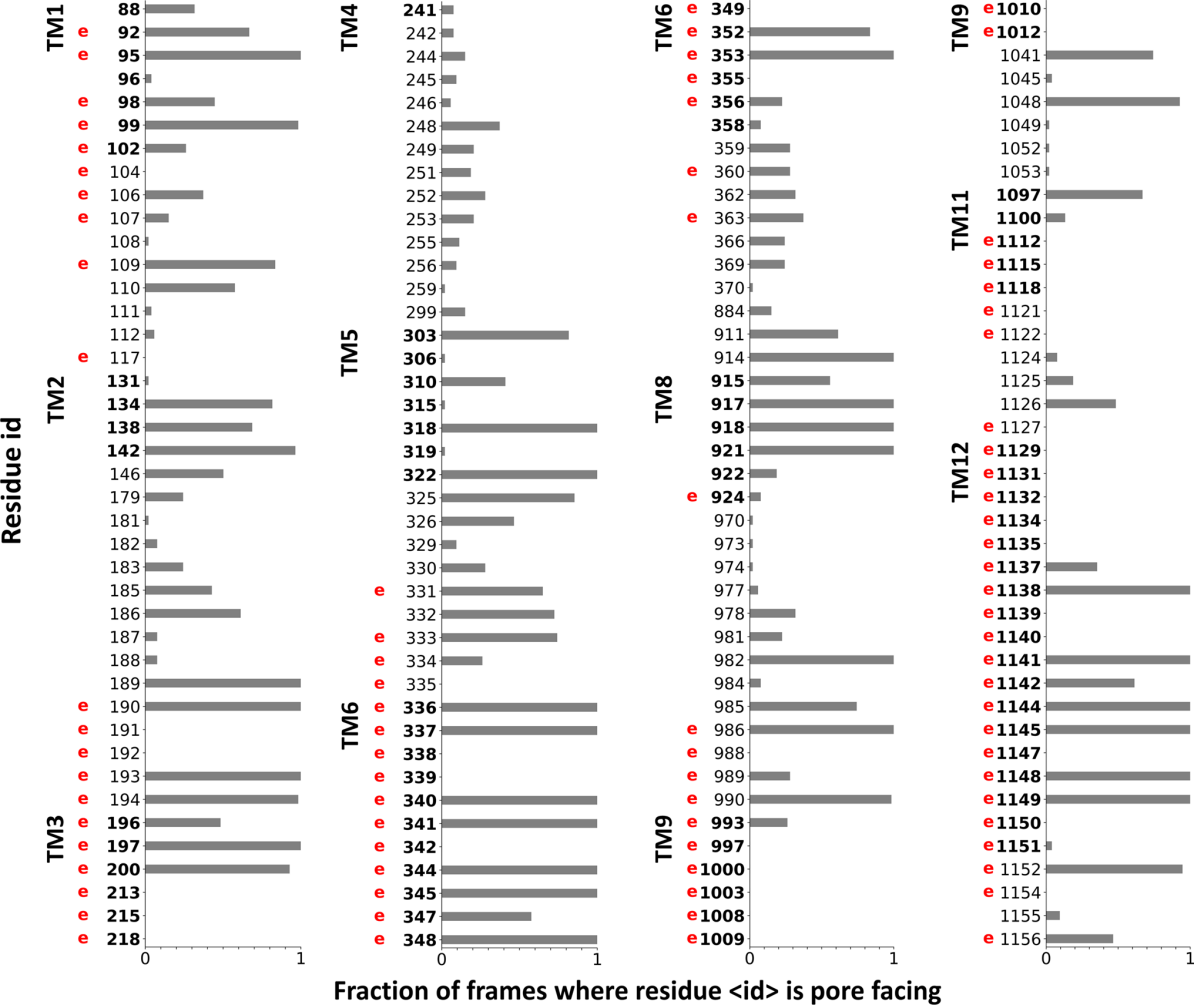
Comparing channel-lining residues identified in simulations to experimental data. Residues interacting with the Caver spheres were counted from all open frames (*n* = 54) and normalized. Only residues lining the channel based on in silico or in vitro data, are indicated in the plot. A letter “e” in red was placed at the residue number (human numbering) to indicate that the residue had been shown by laboratory experiments to influence the CFTR chloride conductance. Bold numbers indicate amino acids located in the TM region

In order to validate our simulations, we related the identified channel-lining residues to locations, which have been experimentally identified as potential residues facing the pathway or playing a role in the chloride conductance [43, 44]. We counted the residues in the 54 open conformations, which were closer than 3 Å to any points of the detected pathways (Caver spheres). The normalized values are plotted in Fig. 2 and experimentally determined channel positions are labeled with a letter “e”. There are numerous residues, which are equally indicated to face the tunnel by both experiments and our simulation. Some of the experimentally identified residues, such as those in TM9, do not participate in the channel formation in our in silico studies. These residues most likely act through allosteric mechanisms, since TM9 is located laterally distant from the putative tunnels. There are also amino acids in TM2, TM5 or TM8, which face the channel in our simulations, but we were unable to find experimental evidences if they are similarly located in vivo.

**Fig. 3:**
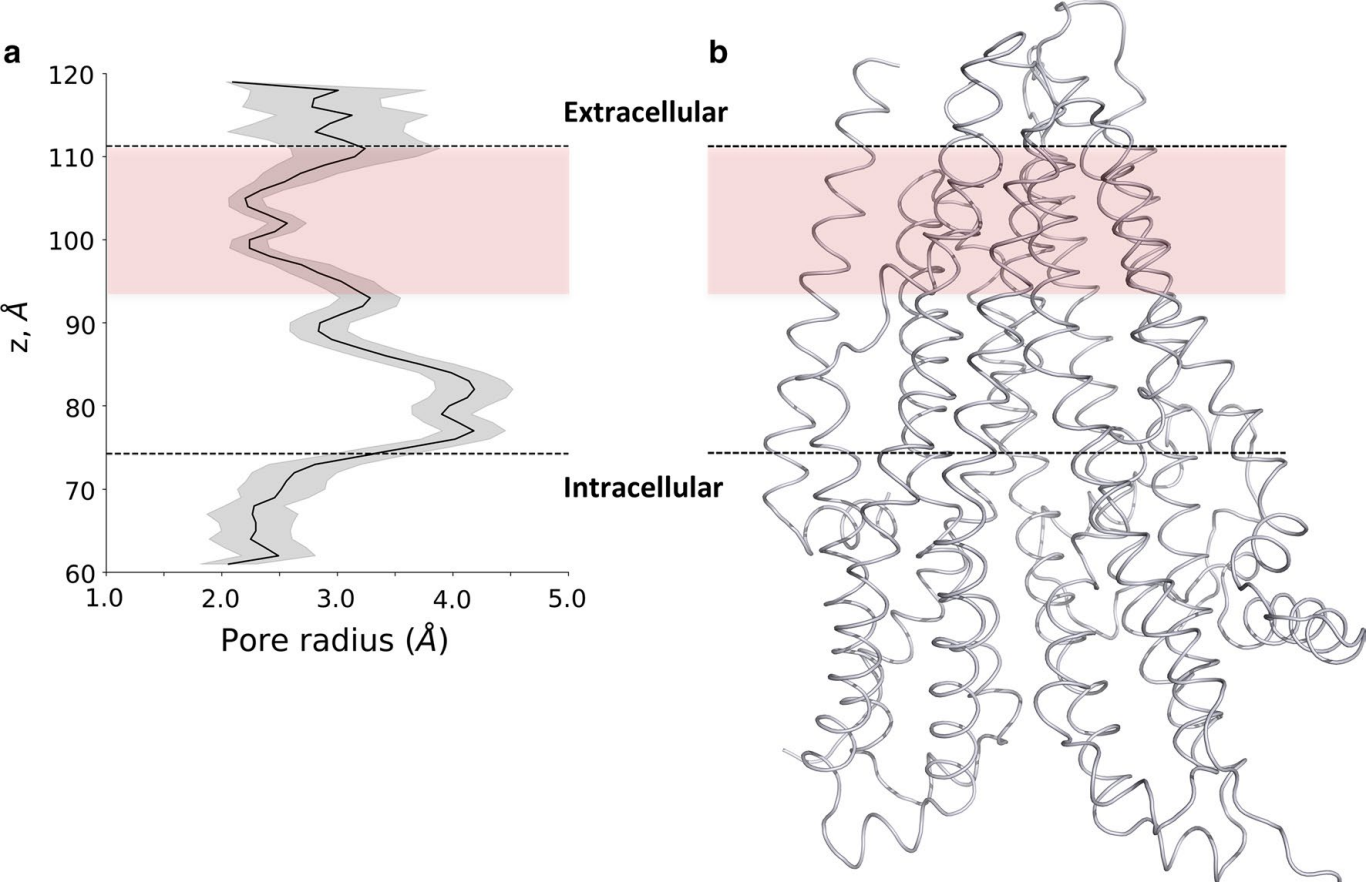
The chloride channel profile. **a** The Caver sphere radius from all open channels was averaged over *z* coordinates. The large inner vestibule is indicated by large values (up to 4 Å), while bottleneck regions in the extracellular leaflet and in the intracellular region are characterized by radius values between 2 and 2.5 Å. The bottleneck in the extracellular membrane leaflet is depicted by the light coral box area. **b** The 3D structures of transmembrane domains are shown in the context of the channel profile

To assess the structural characteristics of the pathway and the possible presence of bottleneck regions, we plotted the average radius of the channel along the *z*-coordinate of the simulation box (the *z* axis is closely perpendicular to the membrane bilayer and the protein) (Fig. 3). The intracellular entry (*z* = 50–75 Å) displayed several critical low values, while in the next region (*z* = 75–95 Å), considered as a large vestibule based on experiments [43–47], the profile showed large radius values. The region between 95 and 110 Å provided bottleneck characteristics with low values close to the radius of chloride ion. Residues at positions Q98, P99, and L102 in TM1, I344 (M in zCFTR) and V345 in TM6, and N1138 (Q in zCFTR) in TM12 had previously been experimentally identified as channel forming residues. In our simulations, these residues were located in the inner vestibule [43, 45–49] and were found in pore-facing positions. In addition, I336, F337, T338, T339, I340 (L in zCFTR) and S341, which were positioned in the bottleneck region we identified in silico, have been implicated as the selectivity filter by several experimental studies [15, 43, 48].

### Interaction of chloride ions with the channel during simulation

Ions show extensive movements in MD simulations compared to large molecules, such as proteins. Therefore, the contacts of chloride ions with amino acids were expected to provide valuable information on chloride interaction sites. It is important to note that the chloride concentration was 150 mM on both sides of the membrane because of the periodic boundary condition in MD simulations.

**Fig. 4:**
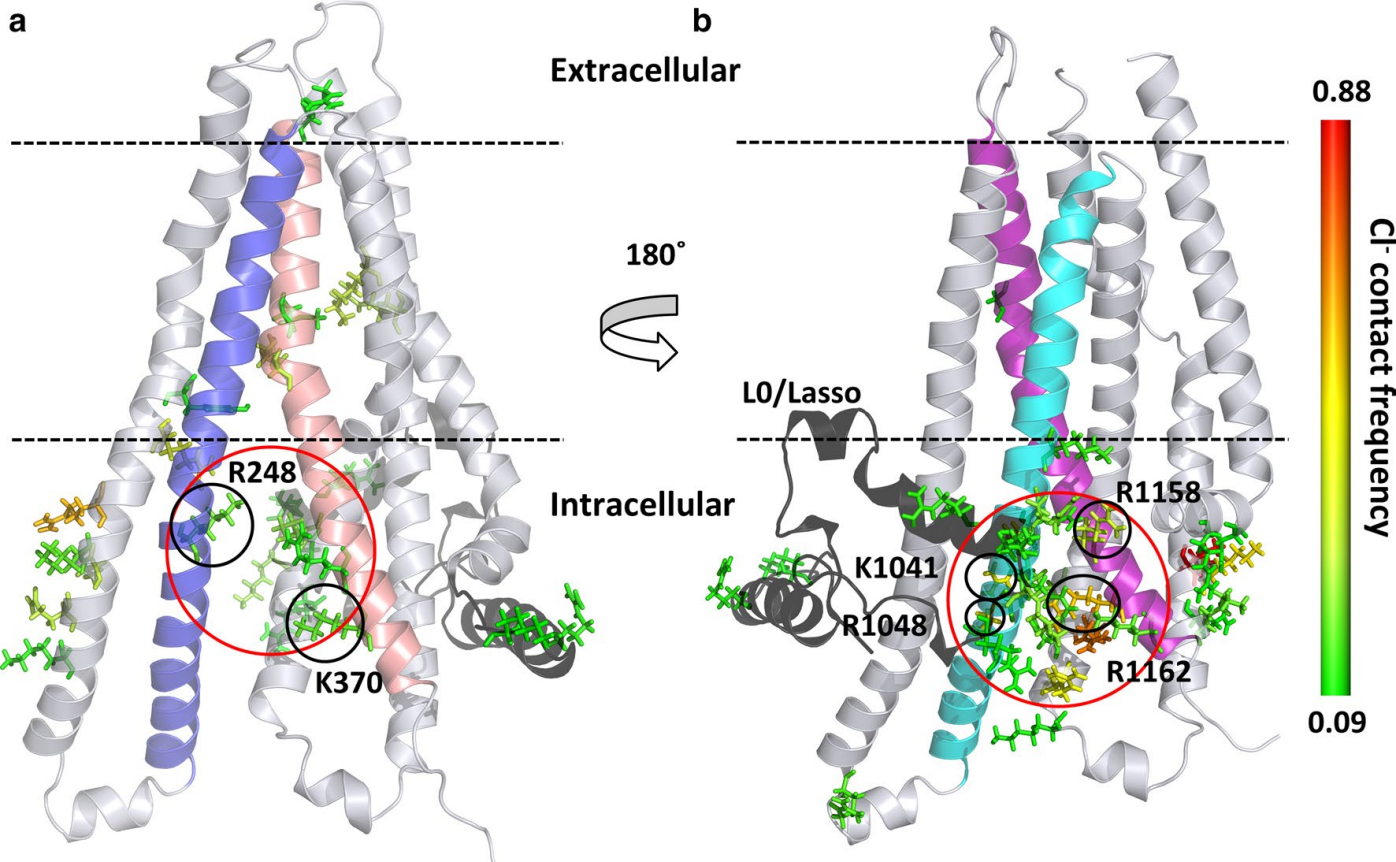
Interaction sites of chloride ions with the CFTR protein. Contact sites are depicted by stick representation of amino acids and color-coded according to the contact frequency from green (low) to red (high). **a** TMD1 and the chloride entry pore at K370, between TM4 and TM6 are shown. **b** TMD2 and the pore close to the L0/Lasso motif (black), between TM10 and TM12 are depicted. TM4, TM6, TM10 and TM12 are colored by blue, salmon, cyan, and purple, respectively. The entry pores are circled (red)

First, we calculated and subsequently normalized the contact map of chloride ions with the protein in all our 100 ns long simulations (*n* = 6). The contact of each amino acid with chloride ions (*d* < 4 Å) was mapped to the structure and colored according to contact frequency (Fig. 4). Intensive interactions could be observed at the entry site defined by TM10 and TM12 close to the N-terminal L0/Lasso region. The frequency of interactions was more pronounced here than at the other entry site defined by TM4 and TM6 near to K370. These interaction sites corresponded to the intracellular pores identified by Caver in our simulations (Figs. 1, S5).

**Fig. 5:**
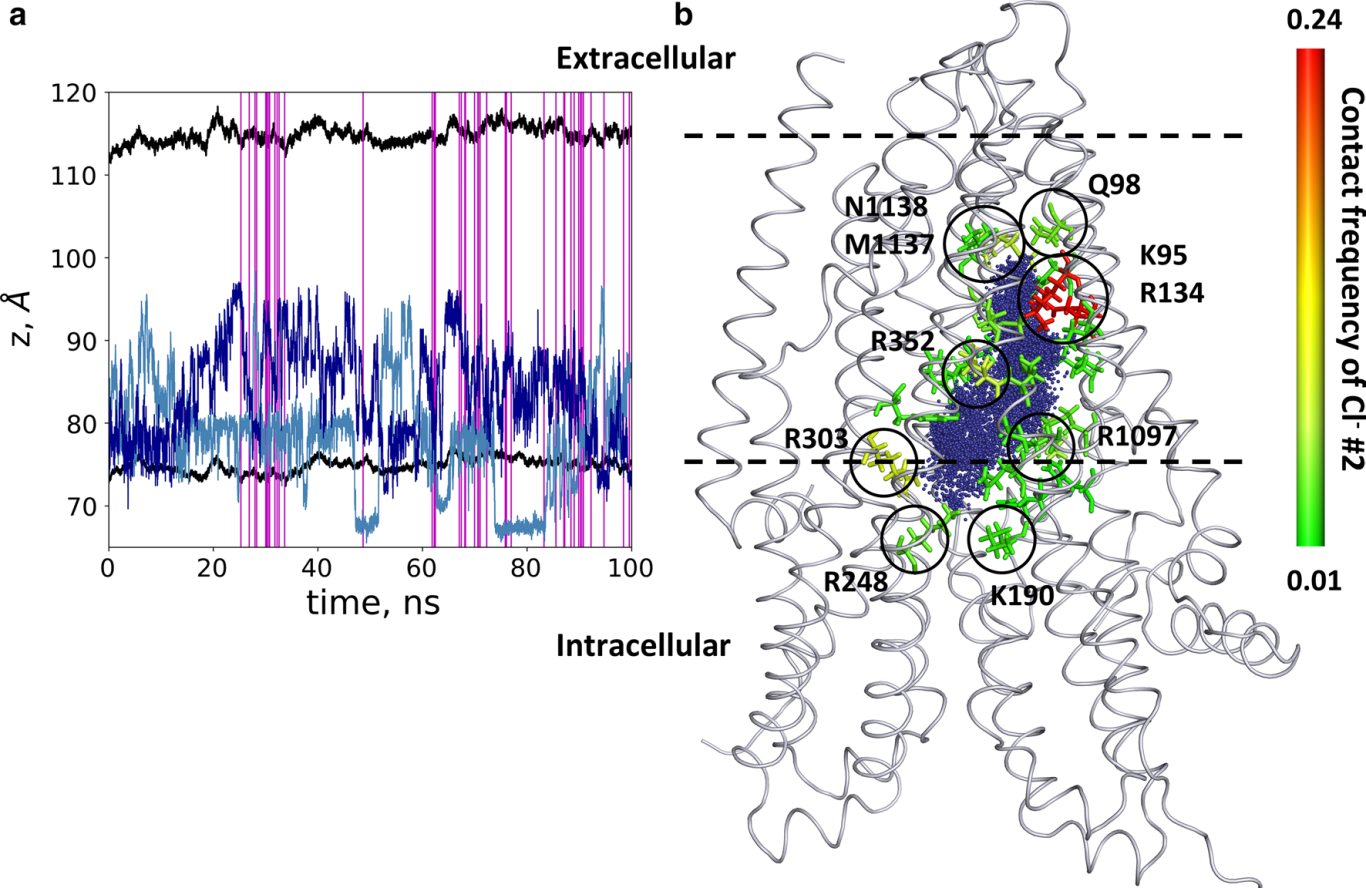
Chloride ions entered the pathway but did not pass. **a** The positions of two chloride ions (#1 light blue and #2 blue), entering the protein, are shown by blue lines. Black lines indicate the boundaries of the membrane bilayer determined by the center of mass of P atoms in POPC molecules. Vertical magenta lines mark frames with an open pathway. **b** The locations of Cl^−^ #2 from every frame are shown with blue dots in the context of the initial structure. The contact frequency of this chloride ion with the protein was projected onto the structure and color coded from green (low) to red (high). Residues with the highest interaction frequencies are K95 and R134 (red)

During the equilibration process, we observed that chloride ions and water molecules filled up the cavity. In the trajectory with open frames, we monitored the position of two chloride ions (labeled as #1 and #2), which entered the inner space of the protein along the membrane normal (Fig. 5). As expected, chloride ions repulsed, and did not remain close to each other. However, these ions did not pass the above described bottleneck region, starting around *z* = 95 Å. Figure 5 also shows that the infrequent open conformations (54 out of 10,000 frames) were distributed over the 100 ns trajectory and were not grouped around a single time point. This observation suggests that we could notice independent opening events in spite of their perceptibility in a single simulation. Because of the low probability of open conformations, most likely none of the chloride ions was in an appropriate position at the moment of an opening, thus we were not able to observe an ion passage. We also calculated the contact frequency of these chloride ions with the pore forming residues (Fig. 5). We observed that chloride ions spent more time close to positively charged amino acids, namely K95, R134, K190, R248 (K in zCFTR), R303, R352 and R1097, facing the large inner vestibule and lining the intracellular opening. Some of these residues had been indicated to be functionally important by experiments, as they affected the chloride conductance in studies using mutant CFTR forms [42, 43, 47, 50–52]. The chloride interaction was less frequent with non-positively charged residues. The two chlorides entering the TM region could reach the bottleneck region but stopped around residues Q98, M1137 and N1138 (L in zCFTR) without passing through the TM region. We also analyzed the interaction and movement of positively charged potassium ions as a negative control. No entry of any potassium into the protein could be detected (Fig. S6).

When we compared the contact maps of open and closed conformations (Fig. S7), residues I344 (M in zCFTR) and N1138 (L in zCFTR) were detected at the extracellular opening of the channel (between TM 1, 6 and 12), participating in closing the chloride pathway. Other residues, namely F337, S341, and L102, surrounding this extracellular pore opening have been suggested to affect channel gating by mutagenesis experiments [15, 43, 45, 48]. Some of these amino acids were located around the observed small opening-up of the ATP-bound human CFTR structure (PDBID: 6MSM), which conformation was suggested to be closer to an open state [20].

**Fig. 6:**
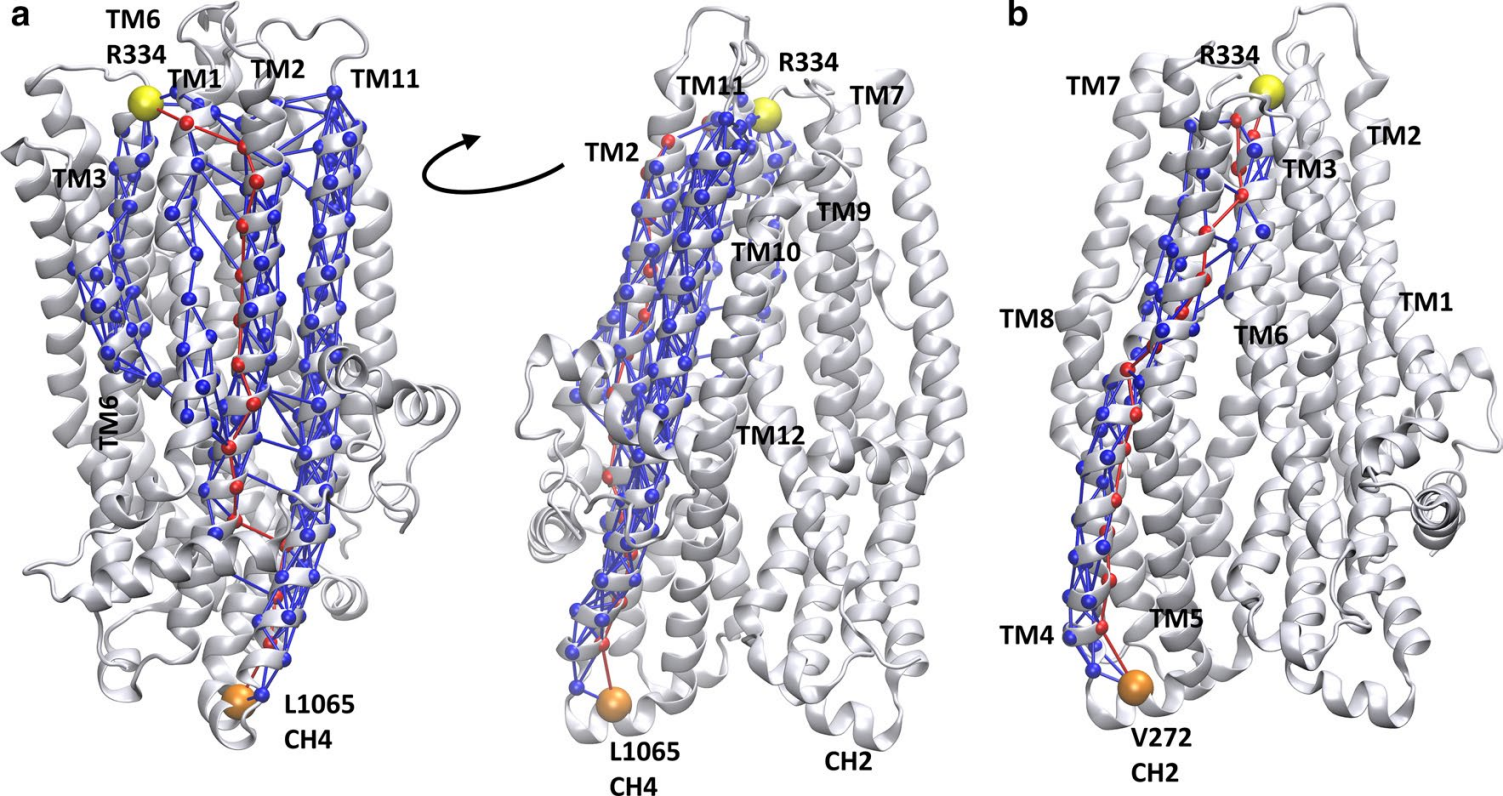
Coupling helix 4 dynamics is strongly coupled to the bottleneck region. Optimal and suboptimal paths in the network of amino acids (spheres), connected by edges (lines) corresponding to correlation in motions, were determined. Number of suboptimal paths between coupling helix 4 (CH4, L1065) and R334 (**a**) is significantly higher than paths from any other coupling helix, such as from coupling helix 2 (CH2, V272) to R334 (**b**). Red: optimal paths; blue: suboptimal paths; orange: source residue; yellow: sink residue

The comparison of the contact maps of open and closed conformations (Fig. S7) revealed regions which have different contacts in open and closed states. These regions are located between the intracellular and extracellular ends of the TM domains including amino acids mainly from TM6 and TM12 and could represent allosteric communication sites for transmitting conformational changes from the nucleotide binding domains to the bottleneck region. In order to characterize the allosteric communication between NBDs and the extracellular ends of the TM helices, we performed a network analysis based on correlations in pairwise residue motion using the VMD NetworkView pipeline [37, 38]. A network was built from amino acids as nodes and their connections were weighted by the level of correlation in their motion. We determined the optimal and suboptimal paths between various “source” and “sink” residues (Fig. 6). The central amino acids (V171, V272, T963, and L1065) of the four coupling helices (CH), which are the main interaction sites between the nucleotide binding and transmembrane domains, were set as “source” nodes. Two amino acids (R334 in TM6 and Y914 in TM8) from the bottleneck residues were selected as “sinks”. We determined the optimal and suboptimal paths in all eight combinations of sources and sinks, since the number of paths correlates with the strength of dynamic coupling. The highest number of suboptimal paths, thus the strongest coupling was observed between the CH4 and sink residues (Table S3). For visual comparison, we plotted L1065/R334 and V272/R334 pathways in Fig. 6. In the first case, several helices (TM1, TM2, TM3, TM6, TM11, and TM12) are included in the paths between the two nodes, while in the latter case only one helix, TM4 and a small region of TM5 and TM6 are involved in the allosteric communication between the intra- and extracellular parts of the CFTR.

### Identification of the barrier of chloride passage using metadynamics simulations

Since the direct passage of chloride ions was not observed during our conventional MD simulations, we performed metadynamics computations to reveal the pathway through the bottleneck region and describe the potential surface of the transition. Metadynamics facilitates the escape from local minima by accumulating history-dependent Gaussian potential on specific reaction coordinates (collective variables, CV) [53]. The frame, in which chloride #2 was most proximally located to the bottleneck region, was selected from the trajectory. This frame was applied in a long, well-tempered metadynamics simulation, where the bias was based on the distance between chloride #2 and the center of mass of four Cα atoms (residues 96, 348, 932, and 1149; human indexing: 95, 347, 924, and 1141). Constraints were applied to prevent the escape of the chloride from the protein towards either the intracellular or the far extracellular directions as described in the Methods section and demonstrated in Fig. S3.

**Fig. 7:**
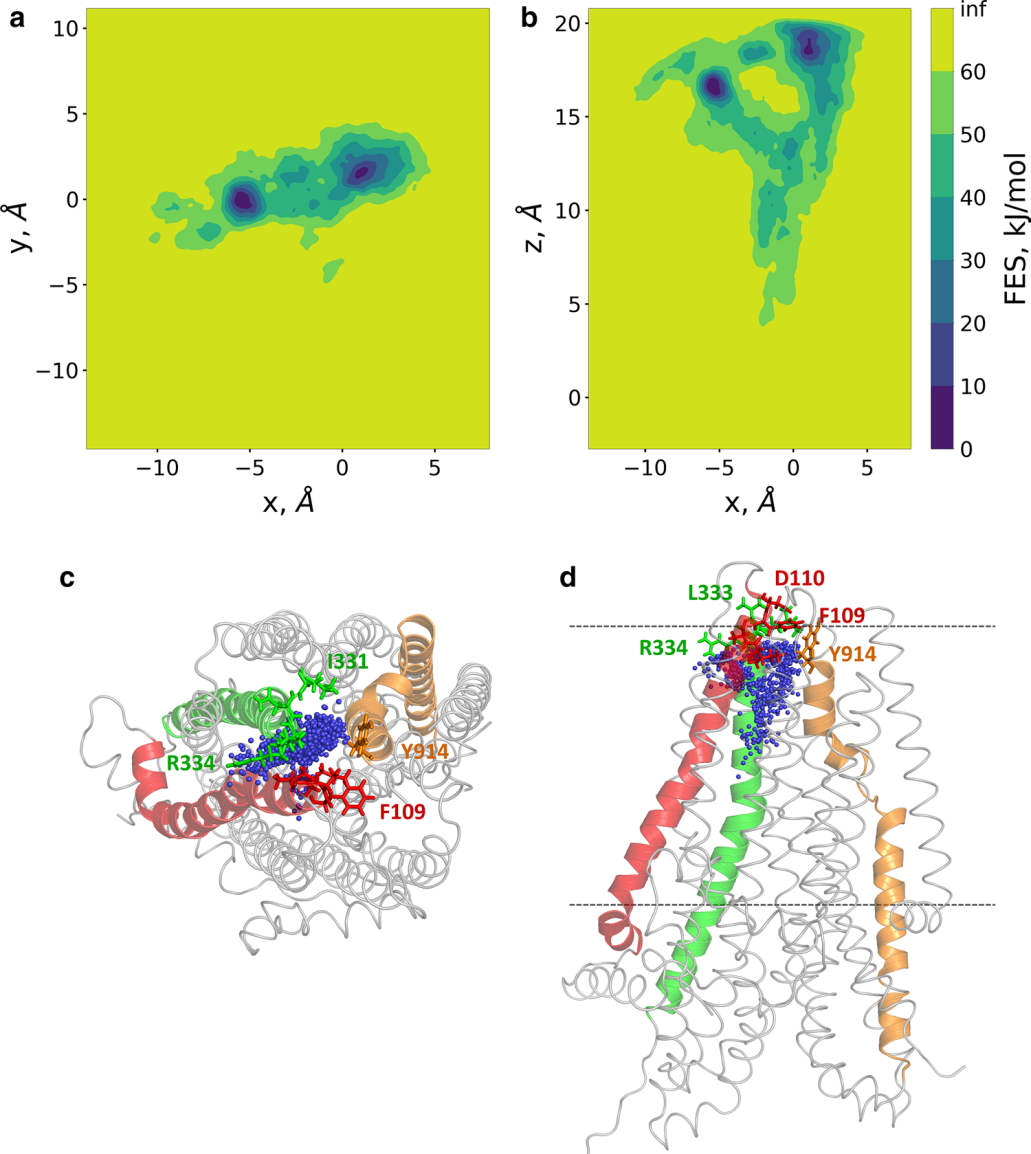
Two routes towards the extracellular regions were detected by metadyanamics simulations. Well-tempered metadynamics simulations were performed to identify potential exit routes of chloride ions and the amino acids narrowing this region of the pathway. The distance between the center of mass of four Cα atoms (a.a. 95, 347, 924, and 1141) and Cl^−^ #2 was used as a reaction coordinate (collective variable, CV). 2D free energy surfaces were calculated along the *x*, *y*, and *z* components of this distance CV and plotted (panels **a**, **b**). Top (panel **c**) and side (panel **d**) views of chloride ions from every 100th frames are shown by blue spheres in relation to the initial structure. TM1, TM6, and TM8 are colored by red, green, and orange, respectively. Sticks represent the following amino acids in the bottleneck region: I106, A107, Y109 (F in zCFTR), D110 (TM1), I331, I332 (N in zCFTR), L333, R334 (TM6), Y914 (TM8)

The well-tempered metadynamics simulation was running for 600 ns allowing convergence (Fig. S4). 2D free energy surfaces were calculated along *x*/*y* and *x*/*z* (Fig. 7a, b) using the *x*, *y*, and *z* components of the distance CV. The *x*/*y* projection, corresponding to a top view from the extracellular space, indicated two deep minima, which were revealed as two extracellular exit sites by the *x*/*z* projection (a side view). This *x*/*z* projection showed that the path split after the bottleneck region and no significant barrier was present along these routes. Amino acids located towards the extracellular side of the bottleneck region and interacting with the chloride ion, include I106, A107, Y109, D110 from TM1, I331, I332 (N in zCFTR), L333 R334 from TM6 and Y914 from TM8 (Fig. 7c, d).

## Discussion

Our computational studies revealed several novel aspects of CFTR chloride channel function, which may prove important for future studies, since to date no open chloride pathway has been identified at the atomic level in spite of recently published cryo-EM structures. Although Das et al. have generated structural models combining experimental data with extensive molecular modeling, their structures were highly different from any known ABC protein structures [54]. Hoffman et al. performed metadynamics simulations biasing the extracellular region of the central helices, applying their homology model that exhibited differences from recent experimental CFTR conformations [6]. In spite of the differences, their results rationally suggest several implications regarding the mechanism of CFTR gating (see below).

Although the cryo-EM structure (PDBID: 5W81) was determined under activating conditions, it did not exhibit an open pathway and it was regarded as a transiently closed conformation. We performed equilibrium simulations to observe the channel opening process with the intention of providing conformations sufficient for chloride conductance. Both, the number of trajectories with open conformations and the probability of channel opening along a trajectory were found to be low, namely 1 out of 22 trajectories and 54 out of 10,000 frames, respectively. This low number of events can be explained by recent electrophysiological data, since Zhang et al. demonstrated that zCFTR exhibited more than tenfold lower open probability when compared to the human CFTR [55]. Importantly, the sampling of the pore forming residues in our simulations corresponded properly to experimental data (see details in “Results”). Most of the observed discrepancies between our simulations and experimental results may originate from allosteric effects. A trivial example of allosteric effects is TM9, which is located laterally distant from the central pore. Therefore, the residues in this helix cannot participate in the chloride pathway, although some of the residues have been indicated by experiments to influence conductance. Similarly, allosteric sites in ABCG2 have been identified by interpreting functional experiments combined with structural data [56]. Such examples strongly suggest that careful analysis and discussion of contradictory biochemical and structural data are important and can become a rich source for identifying allosteric interactions at a high resolution, promoting drug target selection. The other type of discrepancies, where in silico modeling revealed new components of the ion pathway (e.g. TM2, TM5 or TM8), could prompt further mutation experiments. The intriguing concordant data of experiments with hCFTR and in silico simulations with zCFTR suggest that the main conformational changes of these two related proteins associated with channel function may be similar in spite of the evident differences in channel opening probability and conductance [55]. This similarity is also supported by the 54% amino acid identity level with an additional 29% similarity of the zebrafish and human sequences. In addition, the structure of zCFTR determined under activating conditions (PDBID: 5W81 [17]) is highly similar to the more recently published structure of hCFTR (PDBID: 6MSM [20]) solved under similar conditions (RMSD = 1.8 Å). The difference in sequences may result in a stronger interaction of residues in the closed conformation of zCFTR leading to decreased probability of open conformation when compared to hCFTR.

Despite the similarities between experimental and in silico data, several questions remain open concerning channel gating. While several intracellular portals have been indicated by both experimental and computational studies [6, 42], in the light of the structure only one entry site around K370 was favored [17] and the other one at TM10/TM12, in the vicinity of the L0/Lasso motif was neglected. This shift in attention most likely occurred since a cysteine at the position of one of the many positively charged amino acids around this TM10/TM12 pore did not react with methanethiosulfonate (MTS) reagents [42]. Importantly, K1041 and R1048 at this lateral pore are indicated by the same study to play an important role in the electrostatic attraction of chloride ions [42]. We determined low radius values for the channel profile at the intracellular pore region suggesting that the entry point may be selective not only for charge (Fig. S6), but also for size. We were unable to detect chloride entry into the CFTR channel from the extracellular side that could have been attributed to the extracellularly located bottleneck region. The low interaction frequency of chloride ions with amino acids at the extracellular portal and the lack of a well-defined positively charged patch of residues in this extracellular region suggest an important role for EL4. This loop, which was not present in the simulations as it was non-visible in the cryo-EM structures, involves K892 and R899 residues. These positively charged amino acids may attract chloride ions in the area of the extracellular mouth of the protein in case of flow from the extracellular to the intracellular direction.

The intracellular pores are indicated as the first selective part of the channel, since they are narrow and likely provide selectivity for negatively charged ions because of the surrounding positively charged amino acids (Fig. S6). The second level of selectivity and potentially the limiting factor of chloride conductance were present at the bottleneck region in the extracellular membrane leaflet (Fig. 3). The diameter of the pore at this region in the open conformations during our simulations only slightly exceeded the size of the chloride and the diameter in the closed conformations (Fig. S7). This small diameter difference may not be sufficient to allow the passage of the chloride ion surrounded by a hydrate shell. Under physiological conditions, this shell can be weakened by the negative membrane potential, which cannot be modeled in our MD simulations [57]. Although the protein structure was not constrained in an open conformation during our metadynamics simulation, the chloride ion visited both sides of the bottleneck region. Biasing the location of the chloride ion led to a gentle pushing of amino acid side chains and helices by the biased ion. These events were difficult to track because of the subtle conformational differences that were sufficient to provide an open tunnel (Fig. S7). The open state of one of the routes seems to be controlled by the distance between TM1 and TM6, which helices have been shown crucial for gating [15, 43, 45–49].

An intriguing question in the gating mechanism of any channel is, how the conformational changes in the regulatory regions drive the opening of the TM helices. Despite the low number of open conformations, our contact map analysis revealed two hot spots, which were located asymmetrically in the two TM domains, between the NBDs and the bottleneck region along a putative allosteric pathway (Fig. S7). This asymmetry is not unexpected, as the CFTR structure itself is not symmetric and the TM helices are arranged differently compared to helices of other ABC transporters [17, 54]. However, the highly uneven role of coupling helices and pairs of TM helices in dynamic coupling between the NBDs and the extracellular pore region is astonishing (Fig. 6). Coupling helix 4, which is in contact with F508, exhibits an order of magnitude larger number of suboptimal paths towards the bottleneck region compared to all other three coupling helices. This high level of coupling is especially striking, as coupling helix 4 and F508 are in the vicinity of the degenerate, non-hydrolytic ATP site with a tightly bound ATP molecule [58–60]. We propose that the importance of this degenerate site with the stably bound ATP lies in the stabilization of the above mentioned crucial allosteric pathway. The ATP molecule bound to this degenerate site may be structurally indispensable for gating, thus should not be hydrolyzed at a high rate, in order to maintain a stable conformation of the interface between NBD1 and coupling helix 4.

Interestingly, lipid molecules participated in forming the wall of the chloride channel pathway close to the bottleneck region which feature was clearly caused by the helix break in TM8. Although this seems peculiar, the influence of various lipid molecules on CFTR function has been experimentally demonstrated and it is under active investigations. The observed ATPase activity of CFTR has been very low compared to ABC transporters [61, 62]. This phenomenon was anticipated  since the regulation of a channel function is not expected to consume comparable amounts of ATP to those required by active transport processes. However, a recent study described that phosphatidylserine promoted the ATPase activity of CFTR to a level comparable to other ABC transporters [24]. Chin et al. detected both, increased CFTR activity and potentiation in the presence of various lipids [23]. Some of these studies also employed in silico methods to reveal binding sites and to characterize the mode of lipid action. Microsecond MD simulations revealed lipid binding sites in functionally important regions, which may also play a role in conformational transitions. These authors [23] observed preferential binding of phosphatidylserine at helices (e.g. TM4, TM6, and TM10), which participate in forming the intracellular pores. According to our results, at least one of the pathways at the bottleneck region can likely be the target of lipid molecule interactions (Figs. 1, 7). POPC interactions in this region, around the TM8 kink were also detected by Chin et al. [23] and lipid perturbation in the same area was observed in simulations by Corradi et al. [22]. These suggest a hot spot around the TM8 kink, whose conformational transition towards an open state may be influenced or mediated by lipids.

In summary, we described here the chloride pathway of CFTR in atomic resolution for the first time. Our results suggest that several intra- and extracellular entry sites may exist, no large conformational changes of the closed structures are required for opening and lipids may influence the channel path directly not only in an allosteric manner. Our study and methodology may help to understand the gating mechanism of wild type and mutant CFTR proteins.

## Electronic supplementary material

Below is the link to the electronic supplementary material.

Supplementary material 1 (PDF 4085 kb)
