# Supplementary material for: Discovering the chloride pathway in the CFTR channel
Source: Cell Mol Life Sci. 2019 Jul 20;77(4):765–78. doi: 10.1007/s00018-019-03211-4 (PMC7039865; doi:10.1007/s00018-019-03211-4)
Supplement: Supplementary file 1 — Supplementary material 1 (PDF 4085 kb) [file 18_2019_3211_MOESM1_ESM.pdf]

Supplementary Material

Discovering the chloride pathway in the CFTR channel

Bianka Farkas, Hedvig Tordai, Rita Padányi, Attila Tordai, János Gera,

Gábor Paragi, Tamás Hegedűs

Figure S1. The structure of CFTR. Numbering of transmembrane helices is demonstrated on the structure used in our simulations (PDBID: 5W81). CH3 and CH4: coupling helices 3 and 4.

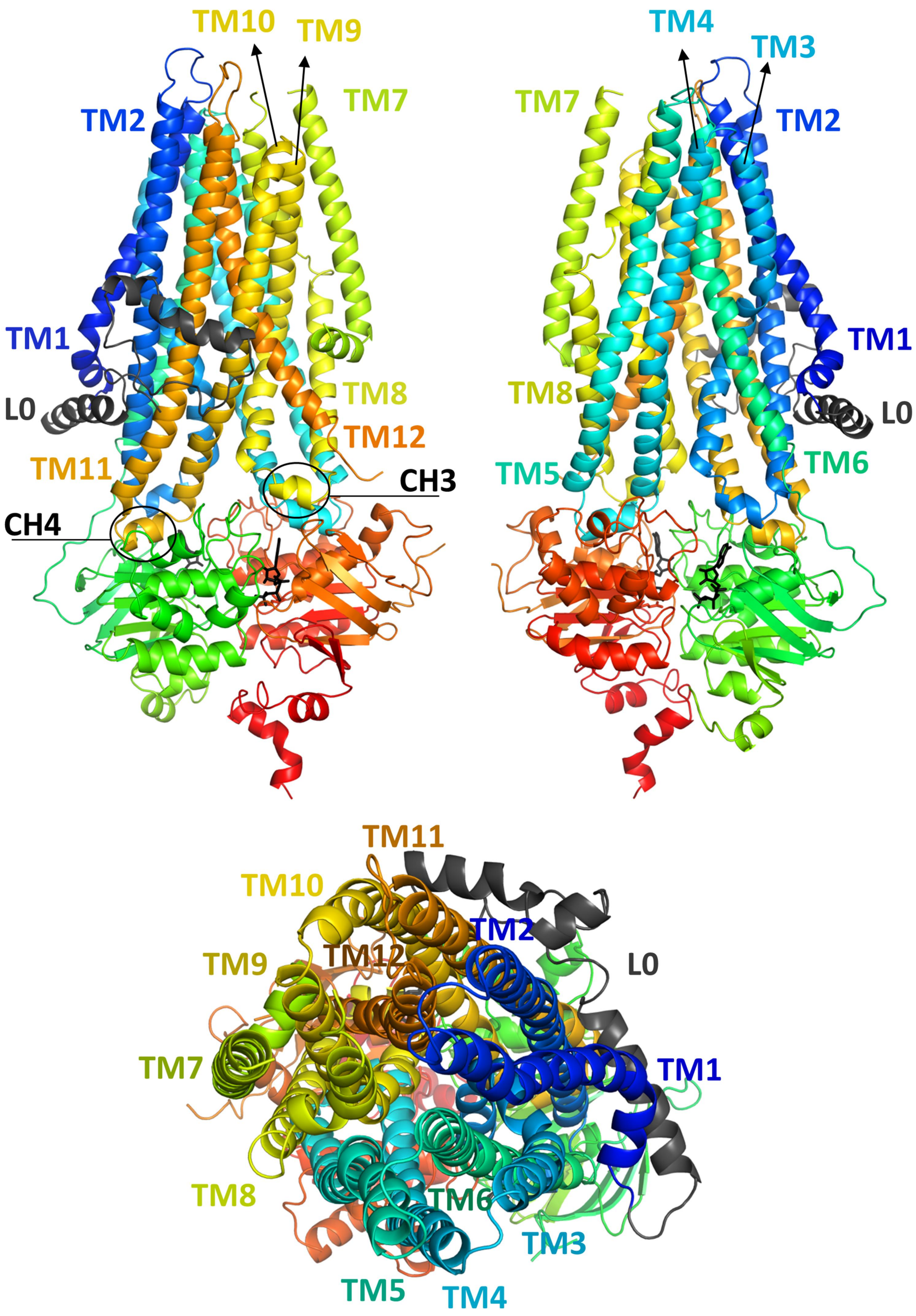

Figure S2. RMSD and R_g plots indicate that our simulations were stable. Root Mean Square Deviation (RMSD) (**a, b**) from the initial protein structure and the radius of gyration (**c**) of the protein were calculated along the six 100 ns long trajectories to characterize the overall quality of the simulations. The simulation #2 with open conformations is highlighted in panel **b**. RMSD values not exceeding 4-5 Å indicate stable simulations with this system size and time scale. In the case of serious instability we expect RMSD values above 6-8 Å [1]. GROMACS tools and matplotlib were used for calculation and plotting, respectively.

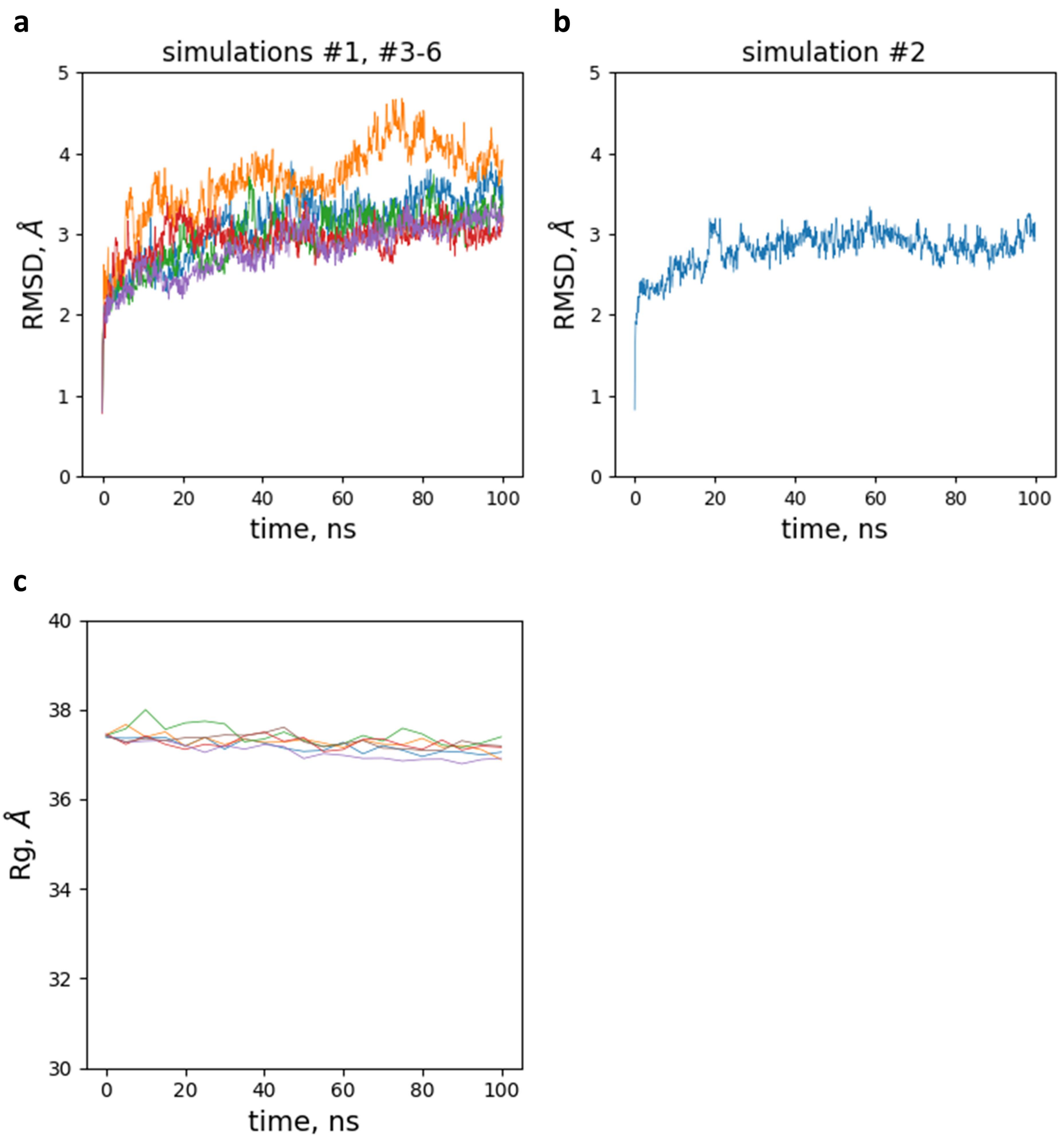

Figure S3. Definition of collective variables and constraints in metadynamics simulations. The distance between the chloride ion (blue circle) and the center of mass (COM, black cross) of four Cα (residues 96, 348, 932, and 1149; human indexing: 95, 347, 924, 1141) was used as a CV which was biased in the metadynamics simulation. The x, y, and z components of this distance CV were applied in the analysis step to recalculate 1D and 2D free energy surfaces (FES). A lower wall at 5 Å (minimum allowed value during the simulation; small black circle) and an upper wall at 20 Å (maximum allowed value during the simulation; large gray circle) were set for this CV for limiting the chloride movement towards the intracellular and the far extracellular space. An angle was also defined based on three points to inhibit the escape of chloride from the region of interest. The angle definition included three points, the COM of POPC P atoms in the extracellular and intracellular leaflets (filled black circles) and the chloride ion (blue circle). The upper wall for this angle (red lines) was set to 80°, which was not highly restrictive. The analysis step suggested that the protein itself contributed to keeping the chloride ion in the region of interest and the application of the walls for the distance CV alone could have been sufficient for our metadynamics simulation. Periodic boundary condition (PBC) does not have an effect of CV calculation, since the PLUMED code takes PBC into account. X: COM of the above mentioned four Cα atoms; d: distance between Cα COM and the chloride ion; x, y, z: components of distance 'd' (distance CV). Two chloride ions are shown for clarity, but only one chloride ion was biased in the simulation.

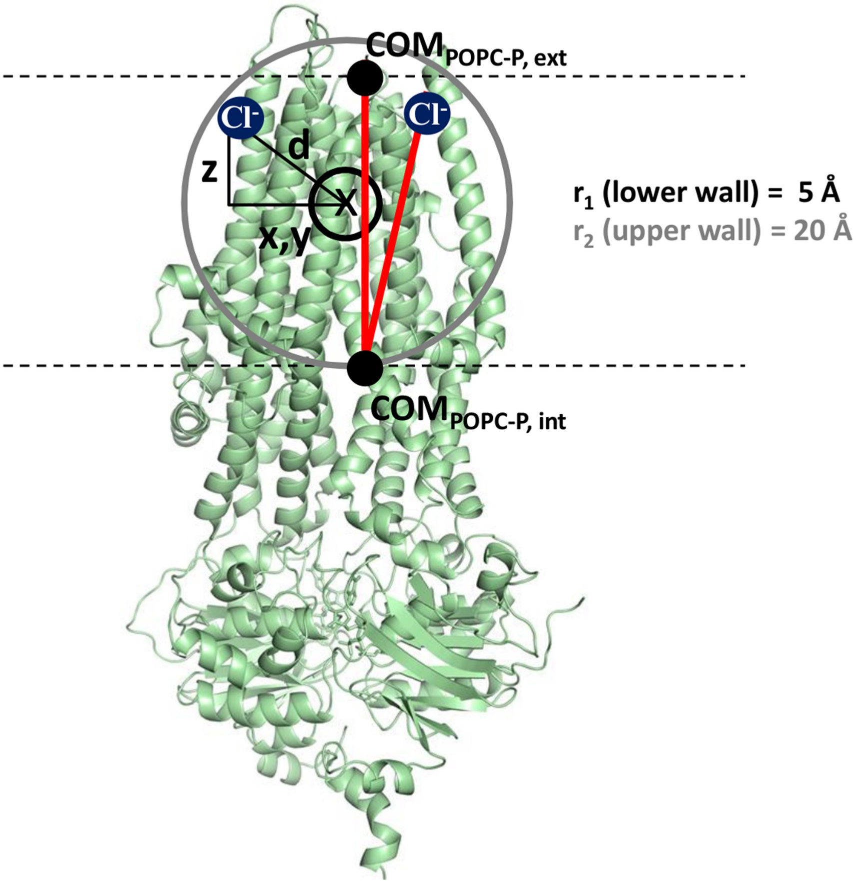

Figure S4. The 600 ns metadynamics simulation converged. The distance between chloride and COM of four Cα was used for bias in the simulation. For analysis the x, y, and z components of this distance were used to recalculate the free energy surface (FES). Here, 1D FES plots along each coordinates at different time intervals, which were calculated by PLUMED tools, show that the metadynamics converged in all three dimensions (lines at later time points became very similar to each other).

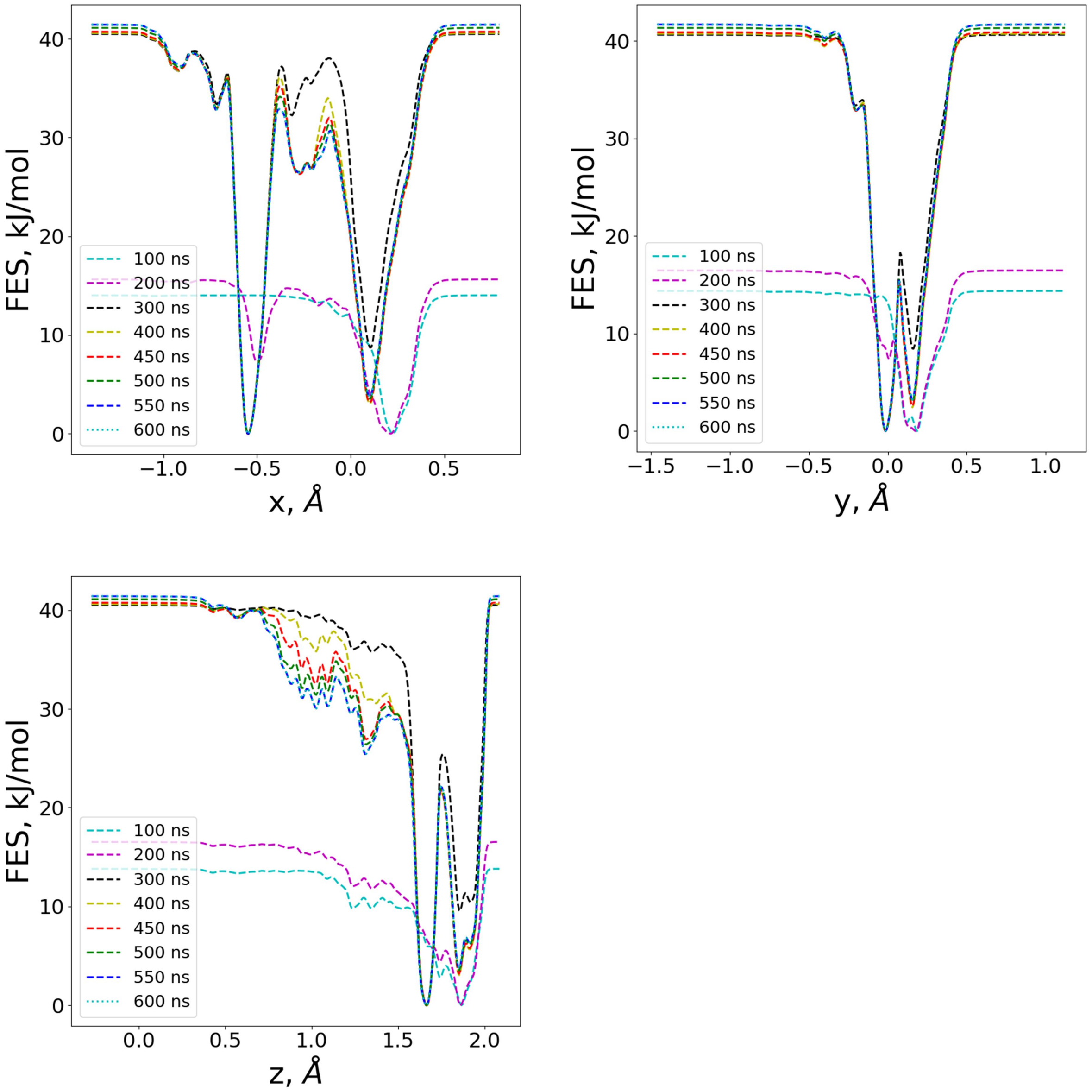

Figure S5. Intracellular entry pores are revealed by Caver. (a) Tunnels in the clusters having TM4/6 entry site. The intracellular parts of the paths, which are shown in Fig. 1, exhibit higher divergence compared to the parts in the bilayer region. (**b**) Channels with intracellular opening between TM10/12 were also detected by Caver, albeit with lower scores. This site is also extensively surrounded by positively charged amino acids (blue sticks). Some of these residues (e.g. K1041 and R1048) have been implied to participate in chloride conductance [2]. The TM10/12 site may have received lower scores because of the presence of L0/Lasso motif, interloping this pore. This intramolecular interaction can be functionally relevant, since L0/Lasso binds syntaxin 1A, which was demonstrated to inhibit CFTR function via direct protein-protein interactions [3].

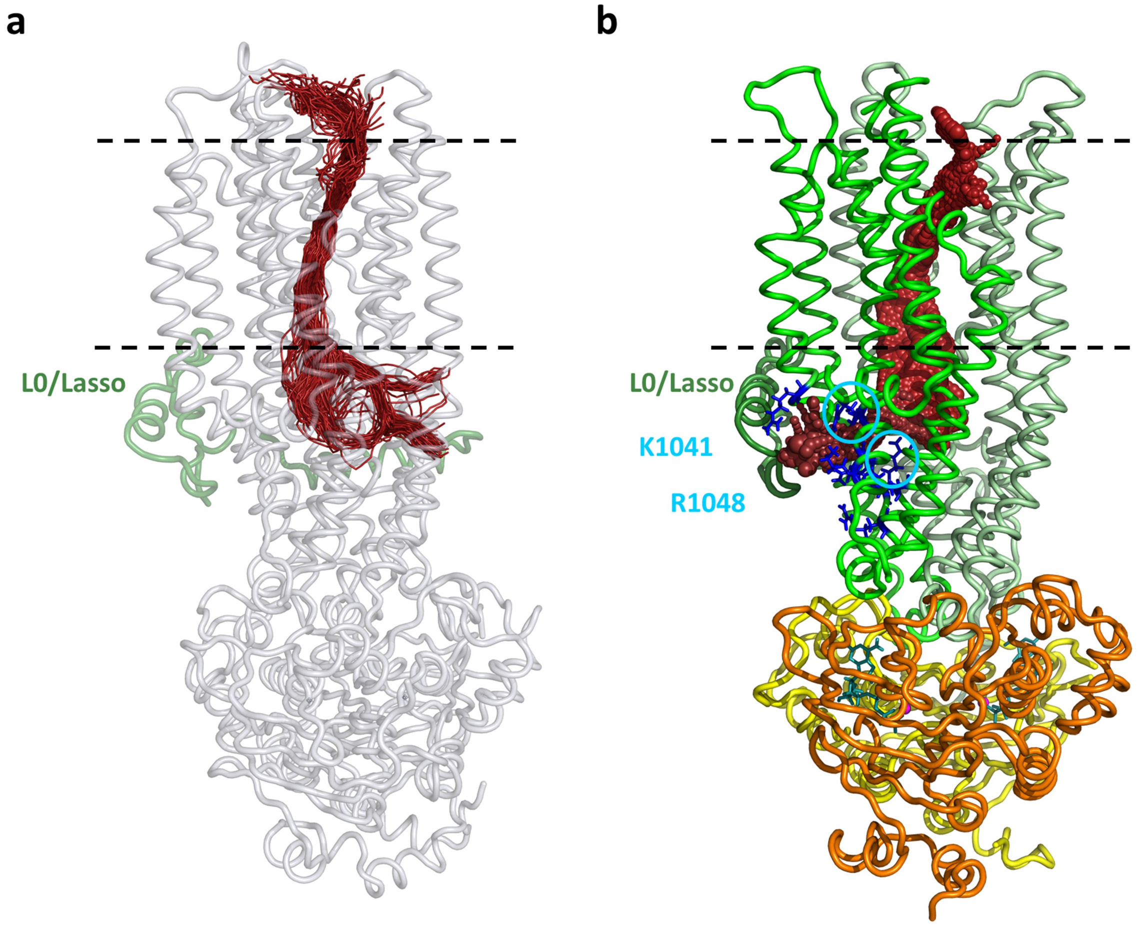

Figure S6. Potassium ions did not enter the inner vestibules. The intracellular openings are surrounded with positively charged amino acids and expected to help in recruiting chloride ions, but it is not known whether positively charged entities can enter into the inner vestibules. To investigate the entry of cations and their interactions with CFTR, we calculated the contact map of potassium ions with the protein in all of our 100 ns long simulations (n=6). The contact of each amino acid with potassium ions (d < 4 Å) after normalization was mapped to the structure and colored according to the contact frequency. These figures indicate that potassium ions interact mostly with extramembranous amino acids and did not enter the inner vestibules in our simulations.

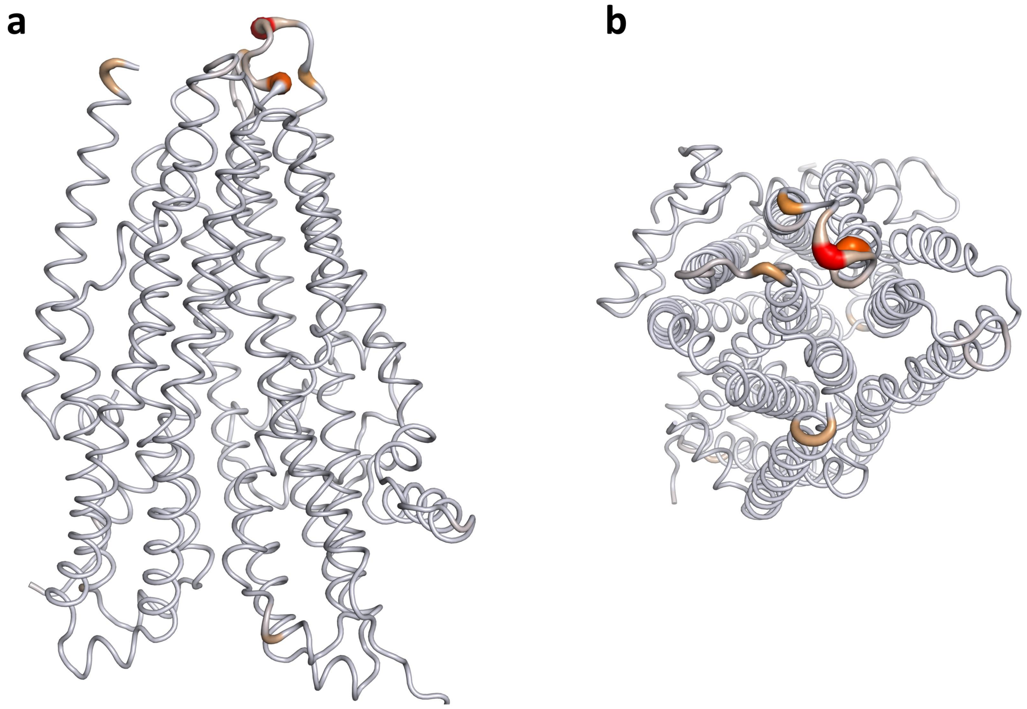

Figure S7. Small changes communicated allosterically to the bottleneck region open or close the gate. (**a**) In order to determine the conformational changes in the bottleneck region responsible for gating, we compared the *frame i* containing open channel and the previous, still closed *frame i-1.* In order to have a broad and quantitative description of the differences, we calculated the protein contact map (heavy atoms in 4 Å) for the closed and open sets of the frames. We subtracted the closed map from the open map and marked the top 10% with the biggest difference in the structure by blue and red lines. Blue and red lines are characteristic for interactions present in closed and open conformations, respectively. For clarity, these lines connect the Cα atoms of interacting amino acids and not specifically their interacting atoms. Amino acids L197, F310, I340, S341, R347, L997, R1048, R1097, and W1145 have interactions in both the closed and open conformations () change their interaction partner during transition. Three regions showing strong and typical changes of interactions can be observed. R1 and R2 regions in the TM domain exhibit more interactions in the closed state, while the bottleneck region (BR) shows interactions mostly specific for the open state. R1 and R2 may be allosteric hot spots for communicating conformational changes between NBDs and the bottleneck region. Contacts characteristic for the closed state include *(1)* mostly TM6 residues and are located in the bottleneck region (I340-V317, I340-S321, I344-G314, and S341-P99), *(2)* TM6 and TM12 interactions in the R1 region (R347- D924, R352-M348, R352-R303, R352-S307, R352-F310, and D1152-T990), and *(3)* TM6 contacts in the R2 region (W356-L197, T360-L197, and T360-G194). Interactions, characteristic for the open state, reside in R1 are provided mainly by TM12 residues (Q1144-M1140, W1145-L997, W1145-S1149, and S1149-L997). Since the major changes in the bottleneck region seem to be lateral and not localized to the pore region, we visually inspected all the closed/open frames pairs. We concluded that the diameter of the pore changes only to a very small extent between open/closed states. A pair of open (**b**) and closed (**c**) structure is shown as an example. Surface representation is used to indicate the blocking volume of amino acids I344 (M in zCFTR) and N1138 (L in zCFTR). Experimentally identified residues affecting the chloride conductance around the exit site are colored green.

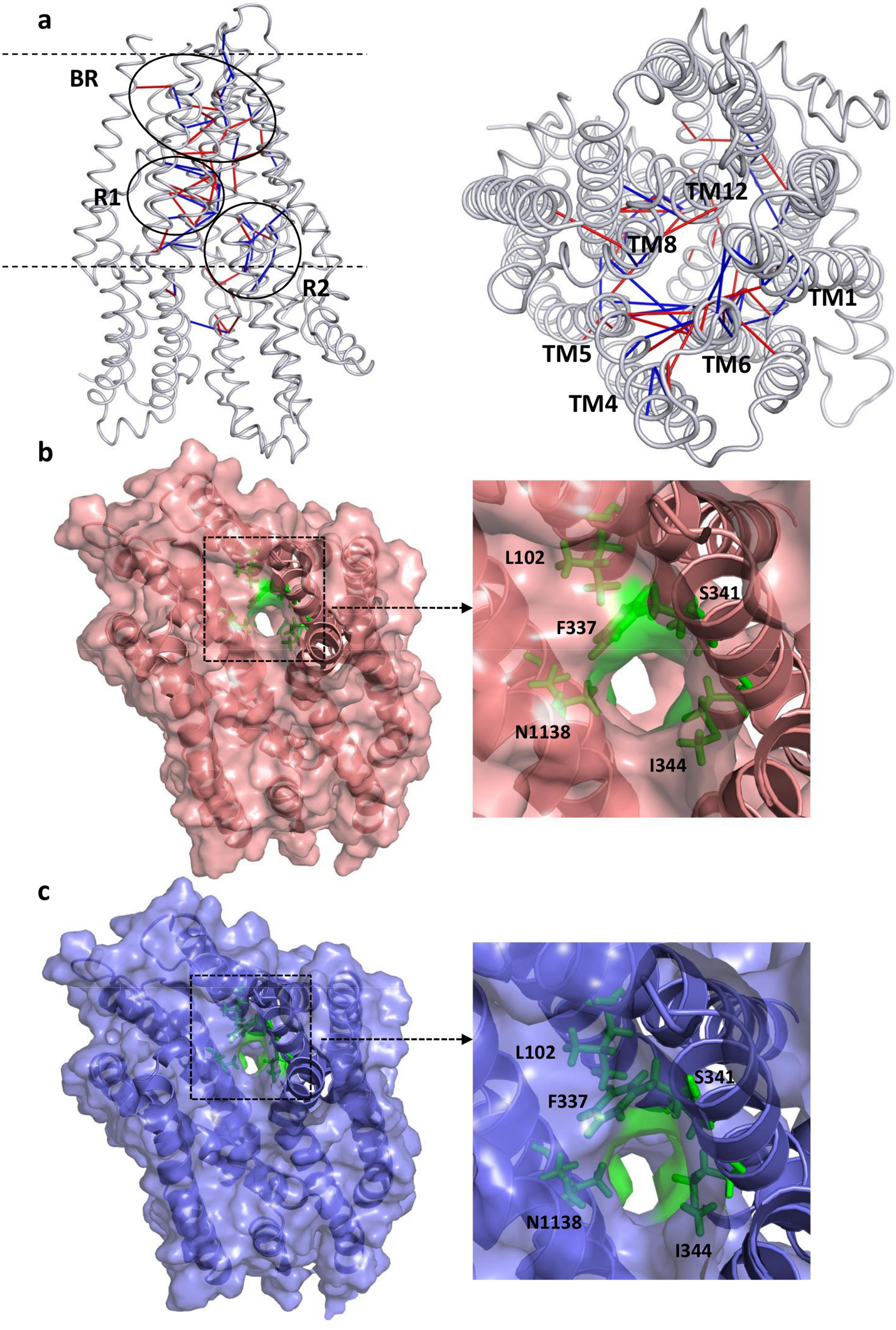

a
BR
R1
R2
TM12
TM8
TM1
TM5
TM6
TM4
b
L102
S341
F337
N1138
I344
c
L102
S341
F337
N1138
I344

Table S1: Simulations performed in this study.

System*	Type	Length**	N
5W81	equilibrium	35 ns	16
5W81	equilibrium	100 ns	6
5W81 chloride #23	equilibrium	20 ns	1
5W81 chloride #23	metadynamics	600 ns	1

* n(all atoms)=197,277; n(POPC)=281; n(Cl^-)=163; n(K^+)=139 ; n(TIP3)=46715

** Total simulation time: 1.78 μs

Table S2: Residues in the bottleneck region separating the inner and outer vestibules.

a.a. #	z-coor, Å	channel radius, Å	channel*	gating**
336	109	2.9	+	+
915	109	2.9		
337	108	2.7	+	+
322	108	2.7		
918	105	2.2		
102	104	2.2	+	
917	104	2.2		
319	103	2.4		
340	103	2.4	+	
99	102	2.6	+	+
318	101	2.5		
341	101	2.5	+	+
921	100	2.2		
922	100	2.2		
98	100	2.2	+	
315	99	2.2		
1138	98	2.4	+	
1137	98	2.4	+	
344	97	2.7	+	
96	97	2.7		
345	95	3.0	+	

* Experiments indicate a.a. with a + mark to influence chloride conductance

** Experiments indicate a role of a.a. with a + mark in gating

Table S3: The number of suboptimal paths between the source and sink residues.

source residue	sink residue	# of suboptimal paths
V171 (172)	R334 (335)	983
V171 (172)	Y914 (922)	516
V272 (273)	R334 (335)	1,639
V272 (273)	Y914 (922)	422
T963 (971)	R334 (335)	558
T963 (971)	Y914 (922)	193
L1065 (1073)	R334 (335)	10,636
L1065 (1073)	Y914 (922)	33,543

References

1. Gyimesi G, Ramachandran S, Kota P, et al (2011) ATP hydrolysis at one of the two sites in ABC transporters initiates transport related conformational transitions. Biochimica et Biophysica Acta (BBA) - Biomembranes 1808:2954–2964. https://doi.org/10.1016/j.bbamem.2011.07.038

2. El Hiani Y, Linsdell P (2015) Functional Architecture of the Cytoplasmic Entrance to the Cystic Fibrosis Transmembrane Conductance Regulator Chloride Channel Pore. Journal of Biological Chemistry 290:15855–15865. https://doi.org/10.1074/jbc.M115.656181

3. Chang SY, Di A, Naren AP, et al (2002) Mechanisms of CFTR regulation by syntaxin 1A and PKA. J Cell Sci 115:783–791
